# Supplementary material for: Pre-hospital causes for delayed arrival in acute ischemic stroke before and during the COVID-19 pandemic: A study at two stroke centers in Egypt
Source: PLoS One. 2021 Jul 14;16(7):e0254228. doi: 10.1371/journal.pone.0254228 (PMC8279320; doi:10.1371/journal.pone.0254228)
Supplement: Attachment — Submitted filename: plos one.docx [file pone.0254228.s002.docx]

Pre-hospital causes for delayed arrival in acute ischemic stroke before and during the COVID-19 pandemic: A study at two stroke centers in Egypt

It was my pleasure to read and review the manuscript: “Pre-hospital causes for delayed arrival in acute ischemic stroke before and during the COVID-19 pandemic: A study at two stroke centers in Egypt” a paper where the different causes of delayed arrival of patients with acute ischemic stroke during a five-month period pre and during de COVID-10 pandemic were analyze and compared.

Before considering this manuscript for publication, I have some comments to add:

Abstract:

Nothing to add

Introduction:

I understand the current situation of pre-hospital care in terms of acute ischemic stroke in Egypt, with the current introduction, but all these paragraphs could be summarized in two, giving special attention to all variables related to patients’ outcome after acute care.

Also, seems relevant to include information regarding the situation of stroke (or medical emergencies) delays after the outbreak of COVID-19 Pandemic in your region or related publications in this topic, as you are comparing both periods, and I cannot see the justification of this behavior in the rationale of your study. This also should be clearly stated in the last paragraph, when adding the main objective of the study.

Methods:

- You have to check the dates in the manuscript because you write that the first COVID’19 case in Egypt was “February 2019”, I assume this was a mistake.

-I suggest to include inclusion and exclusion criteria of your study.

- No information regarding IRB approval protocols are included in your manuscript.

- The survey is a critical part of your study in terms of plausible explanations for delay causes for acute management. I suggest to include the variables and questions for the survey ( a table could be adequate, in this part, instead in the supplementary section).

- Statistical analysis: I suggest to clarify outcome variables for your study, and those included as covariates.

Results:

- I suggest to unify table 1 and 2; both can include the basic variables for the pre-pandemic and trans-pandemic periods
- The section ***Causes of early hospital arrival within window before and during COVID-19 pandemic*** is not clear; I can’t find a clear definition for the word “orientation” in terms of the explanation for causes related to early arrival. I suggest to rephrase this section, as I couldn’t understand it.
- I can’t find a clear association among level of education and NIHSS severity…. Is this a relevant finding?? Or seems like a spurious association from the statistical analysis; if you consider this relevant, I suggest to justify a lot in the discussion section, if not, these results seem irrelevant.

Discussion:

- No clear explanation according to previous literature was done in terms of awareness of stroke as an explanatory variable for delay to consultation, at least from what I can read in this section; this should be analyzed since the first approach from your survey, and who is the responsible for this “not adequate awareness” if present.
- Many sections of the discussion are isolated, and no clear association in terms of explanation for the finding on each section were done. I suggest to follow the same order from your paragraphs, when discussing your findings and respective literature support (references).
- You should add a paragraph stating the limitations of your study.
