# Supplementary material for: Pre-hospital causes for delayed arrival in acute ischemic stroke before and during the COVID-19 pandemic: A study at two stroke centers in Egypt
Source: PLoS One. 2021 Jul 14;16(7):e0254228. doi: 10.1371/journal.pone.0254228 (PMC8279320; doi:10.1371/journal.pone.0254228)
Supplement: Attachment — Submitted filename: Response to Reviewers.docx [file pone.0254228.s004.docx]

***1. There are several grammatical mistakes throughout the manuscript, therefore English editing services are highly required.***

***Done***

***2. When reading the abstract, Background doesn't reflect the purpose of the study; if you are stating that the rationale of the study is to evaluate causes for pre-hospital delay in acute stroke cases seeking for acute revascularization, knowing that your group previously studied this condition seem interesting for the introduction section, but does not explain the reason why you are performing the study. I suggest to re-write te background, and also CLEARLY state that the objective of the present study is to investigate the above-mentioned delay BEFORE and AFTER the COVID-19 outbreak.***

***Done***

***3. Please adhere to Plos One guidelines in terms of tables and figures. Delete colors from columns and rows, and explain abbreviations used. Please erase final markup inside table's text (see table 2)***

***Done***

***4. P value in table 2 refers to...?? There several variables compared among themselves, and only a P value, and I cannot see the correspondance of it; also, if you are intending to show association among both periods, I think that you should add p values comparing each one of the variables***

***Done***

***5. Table 4: I feel that the last column is irrelevant; you could add a superscript explaining the statistical analysis you used. Admission NIHSS result in this table seems odd; I can't understand what you are intending to prove if the comparison is Onset-to-door time and NIHSS... If you are proving that higher of lower NIHSS could increase the delay in terms of consultation, this analysis should be better in a scatter plot, outside this table; if you intend to analyze NIHSS as categories, you should explain since the Methods section, how you manage this variable; as the correlation was negative, I see irrelevant to add this information in a table or a figure.***

***Done***

***6. I don't understand the reason why the questionnaire was generated as a figure; It's a table, and this could be sent as a normal Word format document.***

***Done***
